# Isolated hemangiopericytoma of the conjunctiva

**DOI:** 10.1016/j.ajoc.2022.101308

**Published:** 2022-01-20

**Authors:** William Edwards, Caroline Craven, Joseph Ryan Turner, Hans Grossniklaus, Jill Wells

**Affiliations:** a University of South Alabama College of Medicine, 2451 USA Medical Center Dr, Mobile, AL, USA; b Emory Eye Center, Emory University School of Medicine, 1365 Clifton Rd B, Atlanta, GA, USA

**Keywords:** Conjunctiva, Hemangiopericytoma, Tumor

## Abstract

**Purpose:**

To report a unique presentation of hemangiopericytoma and discuss the clinical course, pathological features, and management of this tumor.

**Observations:**

An otherwise healthy 54-year-old Caucasian female presented with a painless conjunctival mass. The lesion gradually enlarged over a three-week period and was unresponsive to corticosteroid treatment. The mass was surgically removed, and histopathologic findings were consistent with hemangiopericytoma.

**Conclusions and importance:**

Conjunctival hemangiopericytoma should be considered in patients with conjunctival lesions unresponsive to medical management. Surgical excision is diagnostic and therapeutic and is the strongest predictor of clinical course. Incompletely excised lesions are at a greater risk of local recurrence and subsequent metastasis. Given the neoplasm's malignant potential, patients should be followed in the outpatient setting.

## 1 Introduction

Hemangiopericytoma is a rare, vascular neoplasm that originates from the pericytes surrounding capillaries.^1^ They are well documented in literature as soft tissue tumors of the musculoskeletal system. While most ophthalmic cases occur in orbit, there are only four cases of isolated hemangiopericytoma of the conjunctiva reported with the most recent dated in 1994.^2^, ^3^, ^4^

## 2 Case report

**Fig. 1:**
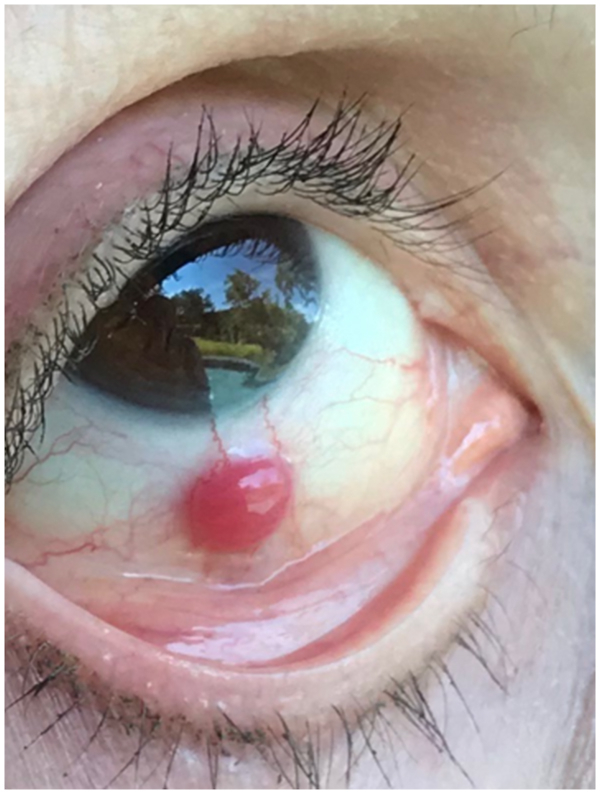
Patient at presentation with inferior bulbar conjunctiva lesion.

A healthy 54-year-old Caucasian female with no known primary cancer presented with a three-week history of a small, non-painful, firm, non-mobile, vascular mass on the bulbar conjunctiva inferiorly (Fig. 1). Prednisolone acetate drops were initiated and with no response after one-month excision was performed. Follow up at one year showed no recurrence and very faint conjunctival scarring with no adhesions or symblepharon.

Microscopic examination revealed proliferative spindle-shaped cells with vesiculated, fusiform nuclei and prominent nucleoli arranged in sheets with associated vascular channels, some of which demonstrating a staghorn pattern highlighted with immunohistochemical stains for CD 31 (Fig. 2A). Reticulin staining revealed collagen surrounding individual cells. Immunohistochemical examination was positive for vimentin uniformly, and the vascular channels stained positive for CD 31 and CD 34 (Fig. 2B).

**Fig. 2a:**
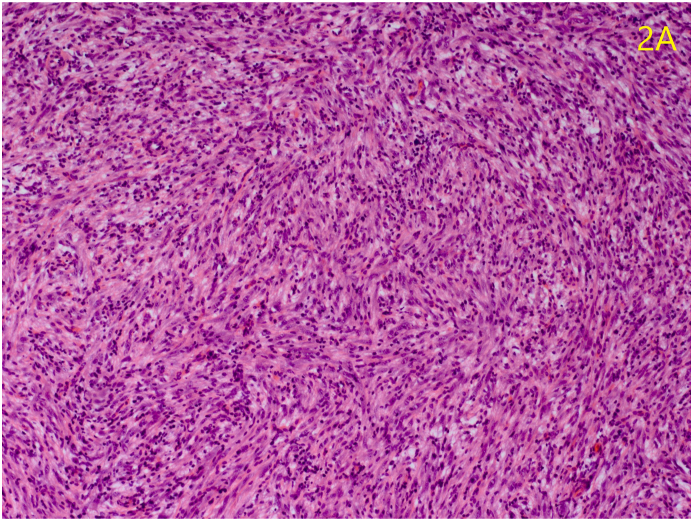
Spindle-shaped cells arranged in sheets (hematoxylin-eosin, magnification 25x).

**Fig. 2b:**
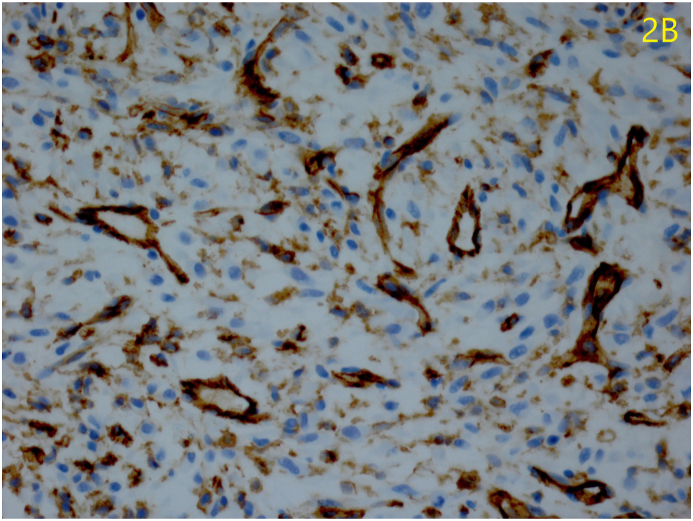
Immunoperoxidase stain CD31 stains vascular channels, demonstrating a staghorn pattern (Immunoperoxidase stain CD 31, magnification x 100).

## 3 Discussion

Hemangiopericytoma is a rare, vascular tumor originating from the pericytes surrounding capillaries.^1^ They present as red or brown painless masses in the fifth decade of life and are usually encountered in ophthalmic pathology as orbital neoplasms.^1^ Its occurrence as a solitary conjunctival mass is very rare, with only four other cases reported in the literature.^2^, ^3^, ^4^

Hemangiopericytomas are solitary neoplasms that demonstrate a haphazard arrangement of spindle-shaped cells outside the endothelial cell basement membrane.^4^ Under light microscopy, our patient's neoplasm resembles the storiform arrangement of fibrous histiocytomas. Tumors exhibiting features of both fibrous histiocytomas and hemangiopericytomas have been reported, but the latter contains a pronounced vascular network as seen in our case. Reticulin defines this vascular network by staining type III and type IV collagen in the vascular basement membrane and wall. Reticulin is used to demonstrate the hemangiopericytoma cells encasing vascular channels as opposed to lining them like hemangioendothelioma cells would.^1^^,^^5^

Conjunctival hemangiopericytomas present similarly to hemangiopericytomas that originate elsewhere in the body.^1^ Of the reported cases, a painless mass was the initial finding, and four out of five patients were noted to have conjunctival injection. These neoplasms often grow insidiously over months, contrasting that of our patient^2^^,^^3^^,^.^4^

To our knowledge, there are no documented cases of conjunctival hemangiopericytomas demonstrating malignant potential.^2^, ^3^, ^4^ However, hemangiopericytomas are known to invade locally, recur, and metastasize, thus conjunctival hemangiopericytomas could behave similarly. Croxatto and Font^6^ conducted a study of 30 orbital hemangiopericytomas and reported a 30% recurrence rate locally. Completeness of local excision appeared to be the best prognostic indicator as 80% of incompletely excised tumors recurred compared to the 7% of completely excised tumors. Metastasis occurred in 50% of recurrent tumors with the lung and bone being the most common sites of metastasis. Similar findings were reported by Enzinger and Smith^1^ in their study of 106 hemangiopericytomas.

Total local excision is the recommended initial treatment modality. Surgical considerations include ensuring complete excision of any capsule, if present, and maintaining hemostasis. Given the propensity of these lesions to recur, patients should have frequent follow-up. Additional surgery is recommended for local recurrence. Postoperative radiation therapy has been used in conjunction with surgery in orbital tumors, but there is no consensus on its use for conjunctival hemangiopericytomas.^5^

## Patient consent

Written informed consent was obtained from the patient for publication of this case report and any accompanying images.

## Funding

No funding or grant support.

## Authorship

All authors attest that they meet the current ICMJE criteria for Authorship.

## Declaration of competing interest

The following authors have no financial disclosures: WE, CC, JRT, HG, JW.
